# SMARCA2 and SMARCA4-deficiency is associated with a distinct molecular and microenvironmental subtype of esophageal adenocarcinoma

**DOI:** 10.1038/s41598-026-60346-8

**Published:** 2026-07-30

**Authors:** Matteo Montalbano, Karl Knipper, Hans A. Schlößer, Bastian Grothey, Reinhard Buettner, Christiane Josephine Bruns, Thomas Zander, Su Ir Lyu, Alexander Quaas

**Affiliations:** 1 Institute of Pathology, Medical Faculty, University Hospital Cologne, University of Cologne, Kerpener Str. 62, Cologne, 50937 Germany; 2 PhD Program in Precision Medicine, University of Palermo, Palermo, Italy; 3 Pathology Unit, Department of Experimental Oncology, Mediterranean Institute of Oncology, Catania, Italy; 4 Department of General, Visceral, Thoracic and Transplantation Surgery, Medical Faculty, University Hospital Cologne, University of Cologne, Cologne, Germany; 5 Department of Internal Medicine I, Medical Faculty, University Hospital Cologne, University of Cologne, Cologne, Germany

**Keywords:** Esophageal adenocarcinoma, SMARCA2/SMARCA4 deficiency, SWI/SNF chromatin remodeling, *MET* amplification, Tumor microenvironment, Biomarkers, Cancer, Oncology

## Abstract

Dysregulation of chromatin remodeling is a key driver of malignant progression. The SWI/SNF ATPase subunits SMARCA2 and SMARCA4 are essential for chromatin dynamics, yet the clinicopathological and microenvironmental landscape of SMARCA2/4-deficient esophageal adenocarcinoma (EAC) remains insufficiently defined. We analyzed 722 resected EACs from a large Western cohort. SMARCA2 and SMARCA4 status were assessed by immunohistochemistry, and tumors were classified as SMARCA-intact or SMARCA-deficient (complete loss of nuclear expression in tumor cells with internal controls). Molecular co-alterations were evaluated by immunohistochemistry and fluorescence in situ hybridization, including amplifications of *MET*,* ERBB2 (HER2)*,* EGFR*,* PIK3CA*,* MYC*,* MDM2*,* TERT*, and Y-chromosome loss (LOY). Several markers were available from prior works. Digital pathology workflows quantified CAF markers (SMA, PDGFRβ, FAP, Periostin, Tenascin) and immune infiltrates (including CD4, CD8, FOXP3, CD20, MUM1, mast cell tryptase). Overall survival was examined using Kaplan–Meier estimates and Cox regression models. SMARCA2/4-deficient tumors accounted for 11.2% of the cohort (81/722) and were enriched among patients aged ≥ 65 years. SMARCA-deficient EACs showed a significantly higher frequency of *MET* amplification (16.9%), particularly after neoadjuvant therapy. Within SMARCA-deficient tumors, PDGFRβ-positive CAFs, increased plasma cell (MUM1+) and mast cell infiltrates correlated with a favorable outcome, whereas loss of Y-chromosome (LOY) identified an adverse-risk subgroup with particularly poor prognosis. This largest-to-date study defines SMARCA-deficient EAC as a distinct subtype characterized by frequent *MET* amplification and high-risk interaction with LOY, alongside prognostically relevant stromal-immune features, supporting refined biomarker-based risk stratification and therapeutic exploration.

**Supplementary Information:**

The online version contains supplementary material available at 10.1038/s41598-026-60346-8.

## Introduction

Esophageal cancer remains a major global health challenge, ranking as the seventh most common malignancy worldwide. Recent estimates from the GLOBOCAN database report an annual burden of over 510,000 new cases and approximately 445,000 deaths. The disease exhibits a significant gender disparity, with men being affected two to three times more frequently than women^1^. The histological landscape is dominated by two main subtypes: squamous cell carcinoma (SCC) and adenocarcinoma (EAC). While the incidence of SCC has declined due to reduced tobacco and alcohol consumption, EAC cases have risen significantly, especially in the Western world. This increase is largely linked to the prevalence of Barrett’s esophagus, driven by risk factors such as obesity, gastroesophageal reflux disease (GERD), and dietary habits^2,3^.

Despite therapeutic advancements, the prognosis for esophageal cancer remains poor. Treatment typically relies on multimodal approaches, including preoperative radio-chemotherapy (CROSS) or perioperative chemotherapy (mainly FLOT or combined Durvalumab-FLOT), with low survival rates. While early detection of precursor lesions like Barrett’s dysplasia holds promise for improving outcomes, there is a critical need for novel biomarkers to better understand the tumor biology of specific EAC subgroups and to facilitate personalized therapeutic strategies^4^. A fundamental driver of oncogenesis and disease progression is the dysregulation of chromatin dynamics. Central to this process is the SWitch/Sucrose-NonFermenting (SWI/SNF) complex, a key ATP-dependent chromatin-remodeling system. This multi-subunit complex, which is mutated in nearly 20% of all human cancers, regulates gene expression by altering nucleosome positioning. In humans, the complex’s catalytic activity is powered by one of two mutually exclusive ATPase subunits: SMARCA4 (BRG1) or SMARCA2 (BRM). These subunits play overlapping roles in cellular development, differentiation, and stem cell regulation^5–8^. The functional relationship between these paralogs has significant clinical implications. In many cancers, the loss of one ATPase (e.g., SMARCA4) renders the cell dependent on the remaining one (SMARCA2), making selective ATPase inhibition a promising synthetic lethal therapeutic strategy^9,10^. While the simultaneous loss of both SMARCA4 and SMARCA2 is exceptionally rare, it has been observed in a subset of highly aggressive, undifferentiated malignancies across various organs, including the lung, ovary, and gastrointestinal tract. In the context of the esophagus, recent studies have identified SMARCA2/4 deficiency in undifferentiated adenocarcinomas characterized by rhabdoid morphology and diffuse, high-grade features^11–14^. However, the specific clinical and molecular landscape of SMARCA2/4-deficient esophageal adenocarcinoma remains poorly defined.

Previous investigations on this large EAC cohort established the prognostic impact of Y-chromosome loss (LOY)^15^ and the proteomic landscape of *MET-*amplified tumors^16^. However, the potential interplay between these alterations and the SWI/SNF chromatin remodeling complex remained unexplored. The current study aims to bridge this gap by defining the SMARCA-deficient subset not as an isolated finding, but as a distinct entity that integrates *MET* signaling and large-scale genomic instability (LOY) within a specific microenvironmental context. Here, we characterize a large cohort of EACs with SMARCA2 and SMARCA4 deficiency, comparing them to SMARCA-intact EACs. Specifically, we investigate their clinicopathological features, histological growth patterns, molecular co-alterations, and the composition of the inflammatory tumor microenvironment (TME), including different subtypes of cancer-associated fibroblasts (CAFs).

## Materials and methods

### Patient cohort and tumor samples

This study analyzed formalin-fixed, paraffin-embedded (FFPE) tissue samples from a cohort of 722 patients diagnosed with esophageal adenocarcinoma (EAC). All patients underwent either primary surgical resection or resection following neoadjuvant therapy at the University Hospital of Cologne, Germany, from 2013 to 2019. Overall survival was defined as the time from surgery to death or loss of follow-up. Patient data were prospectively collected and retrospectively analyzed. Written informed consent to participate in our tissue bank was obtained from every individual. This study complied with the Declaration of Helsinki and was approved by the local ethics committee (protocol code: 20-1393). Specimens were assessed following the 7th edition of the Union for International Cancer Control. Detailed clinicopathological characteristics are summarized in Table 1. To facilitate high-throughput analysis, we utilized tissue microarrays (TMAs). For 81 patients exhibiting loss of expression of SMARCA2 and/or SMARCA4, a multi-spot TMA was constructed, incorporating up to four tumor cores per patient. To account for intratumoral heterogeneity, samples were strategically selected from the tumor surface, center, and invasive margin whenever feasible. For the remaining 641 patients with intact SMARCA2/4 expression, a previously established single-spot TMA was employed. Cases with missing molecular or immunohistochemical data due to random technical artifacts (e.g., tissue microarray core exhaustion or detachment during processing) were managed using a complete-case approach. Because these data losses were driven strictly by mechanical factors during laboratory processing, they occur at random and do not affect the representative nature of the analyzed cohorts. TMA construction involved extracting tissue cylinders (1.2 mm diameter) from representative donor blocks using a semi-automated precision instrument. These cores were re-embedded into recipient paraffin blocks. Finally, 4-µm sections were cut and transferred onto adhesive-coated slides for further analysis^17^.

### Histological classification

Tumors were stratified into two main architectural groups: tubular and non-tubular. The tubular pattern was defined by the presence of well-to-moderately formed glandular structures (corresponding to Lauren’s intestinal type). The non-tubular pattern was characterized by poorly differentiated morphologies, including solid nests, single-cell infiltration (discohesive) or rhabdoid features (corresponding to Lauren’s diffuse or indeterminate types)^18^. To address intratumoral heterogeneity, a conservative classification strategy was applied: cases with mixed histological patterns were assigned to the non-tubular group, regardless of the percentage of the non-tubular component.

### Markers analyzed using immunohistochemistry (IHC)

IHC was performed on the TMA sections using standardized protocols on the automated Ventana/Roche slide stainer. The expression of SMARCA2 and SMARCA4 was independently evaluated by two pathologists (AQ and SIL) using the following scoring system: SMARCA2 and SMARCA4 protein expression was categorized as ‘Intact’ or ‘Deficient’. Cases showing a weak but discernible and diffuse nuclear signal were categorized as having preserved expression (Intact). Conversely, a case was classified as ‘Deficient’ when showing either a complete absence of nuclear staining in all viable tumor cells or a patchy/heterogeneous staining pattern (defined as a mixture of positive and negative tumor cells within the same core), provided that a mandatory internal positive control (e.g., stromal or endothelial cells) was present^9^. In addition to SMARCA2 and SMARCA4, the cohort was characterized for HER2/neu, Claudin 18.2, MTAP (defining the MTAP-loss EAC subtype), and CK5-CK6 as positive if at least one of both cytokeratins are expressed in more than 50% of tumor cells. These markers have been analyzed in previous projects from our group^19–21^. The positive expression of Claudin 18.2 was based on ≥ 75% of tumor cells exhibiting moderate-to-strong (2+/3+) membranous staining^21^. HER2 positivity was established based on diagnostic criteria adapted for upper gastrointestinal biopsies^22^. Specifically, a score of 3 + was defined by intense complete, lateral, or basolateral membranous reactivity within tumor cell clusters containing five or more cells. Cases showing equivocal, weak-to-moderate (2+) membranous staining in identical cell aggregates were further evaluated via Fluorescence In Situ Hybridization (FISH) to confirm gene amplification, in line with previously validated screening protocols for this cohort^19^.

We also evaluated a panel of stromal and immune cell markers, including PDGFRβ, Periostin, FAP, SMA, and Tenascin to describe CAF-subtypes as well as different immune cell related markers such as CD4, CD8, CD20, CD56, CD66b, CD68, CD163, ERG, FOXP3, MCT, and MUM-1, following the manufacturers’ instructions (for details see Suppl. table S1). A part of these markers has been analyzed in this patient cohort in previous works of our group^16,23–26^. Some markers were only available for a fraction of patients, causing missing data. These data points were not imputed.

We determined the extensive marker specifications on the tumor tissue using software-based methods. The stained slides were digitized using an automated digital image scanner (Leica, Aperio GT450 DX) and analyzed via a customized QuPath (v0.3.2) workflow^27^. For the stromal CAF-markers (SMA, PDGFRβ, Tenascin, Periostin, and FAP), the positive DAB area was quantified, utilizing a resolution threshold of 2.11 μm/pixel and a Gaussian pre-filter (sigma 1, threshold 0.07).

The immune cell infiltration was quantified using an optical density sum detection method (pixel size 0.5 μm). Cell segmentation included a 5 μm expansion from the nucleus, with nuclei defined by a background radius of 8 μm and a minimum area of 10 μm². For analysis, the percentage of positive cells among all cells was calculated. The cut-off between low and high positivity was defined by the median, with median values assigned to the high group.

### Markers analyzed using Fluorescence in situ hybridization (FISH)

EACs are molecularly characterized primarily by the amplification of oncogenes. We therefore identified relevant oncogenes using FISH. Again, some markers were available to us from earlier publications^15,19,28–31^. Tumor tissue was scanned for amplification hot spots using a 63x objective (DM5500 fluorescent microscope; Leica). We assessed the amplification status of *TERT*,* MDM2*,* MET*,* MYC*,* PIK3CA*, and *EGFR* following the manufacturer´s instructions (for details, see the supplement’s table S2). *PIK3CA* amplification was defined as a *PIK3CA/CEN3* ratio ≥ 2.0 or ≥ 5.0 signals per cell^32^. *MET* amplification was characterized by a *MET/CEP7* ratio ≥ 2.0 or a gene copy number > 4^33^. For *MYC*, amplification was defined by the presence of gene clusters in > 50% of tumor cells or a total gene copy number > 6^34^. *MDM2* gene amplification status was established when tumor samples met at least one of the following criteria: a minimum of 50% of evaluated cells dislaying five or more *MDM2* signals, at least 10% of cells harboring 15 or more gene copies, an overall *MDM2/CEN12* ratio of 2.0 or higher, or a mean absolute *MDM2* copy number of six or more per cell^31^. *TERT* FISH analyses were scored as positive for amplification if the target *TERT* signal (green) exhibited at least a two-fold increase relative to the reference centromere signal (red), or if a mean threshold of six or more gene copies per cell was identified across twenty assessed tumor nuclei^35^. For the remaining genes, a ratio ≥ 2.0 or gene copy number per cell ≥ 6.0 was used to define amplification. For cases with equivocal HER2 IHC expression (score 2+), mandatory validation was performed by evaluating twenty tumor cells, counting green *HER2* and orange centromere *17 (CEN17)* signals.

Following recommendations, a *HER2/CEN17* ratio ≥ 2.0 or *HER2* signals per cell ≥ 6.0 in 20 tumor cells established gene amplification, whereas a ratio < 2.0 was considered negative^19^.

*EGFR* status was assessed by FISH using the ZytoLight SPEC *EGFR/CEN 7* probe (Zytomed Systems GmbH, Berlin, Germany), defining amplification as an *EGFR/CEN 7* ratio ≥ 2.0, the presence of gene clusters, or a gene copy number per cell ≥ 6.0 evaluated in 60 tumor nuclei^36^. Y-chromosome status was derived from previous project using probes (Yq12/Yp11.3) (Abbott Molecular, Wiesbaden, Germany); complete loss of both green and orange signals in tumor spots was defined as loss of Y^15^(for details see Suppl. table S2).

### Data analysis and statistics

Statistical analyses were performed using IBM SPSS Statistics (Version 29.0.1.1, IBM, Armonk, USA). p-values below 0.05 were defined as statistically significant. Qualitative variables were compared using the Chi-Square test. Survival outcomes were estimated using Kaplan–Meier curves and the log-rank test.

## Results

### Patient and tumor characteristics

The study cohort comprised 722 patients with EAC, categorized into SMARCA-stable (*n* = 641) and SMARCA-deficient (*n* = 81, 11.2%) groups. SMARCA 2 deficiency was detected in 73 (10.1%) patients, 18 (2.5%) for SMARCA 4, and only 10 (1.4%) showed loss of expression for both proteins. Detailed clinicopathological data are presented in Table 1. Specific subgroup analyses were conducted for SMARCA2 deficiency, SMARCA4 deficiency, and dual deficiency. These subanalyses are presented in Supplementary Tables (Suppl. Table S3, S3a, S4, S4a, S5, S5a and Suppl. Figure S1).

**Table 1:** Clinicopathological features of esophagus adenocarcinoma patient’s cohort according to SMARCA status. Bold print indicates statistical significance. (y)pN: pathological lymph node status (after neoadjuvant therapy), (y)pT: pathological tumor status (after neoadjuvant therapy), L0: No lymphatic vessel infiltration, L1: lymphatic vessel infiltration present, L2: extensive or diffuse lymphatic vessel, V0: No blood vessel infiltration, V1 microscopic blood vessel infiltration, V2: macroscopic blood vessel infiltration.

Clinicopathological features	Total cohort (*n* = 722)	SMARCA deficient (*n* = 81)	SMARCA stable (*n* = 641)	*p* value
	100%	11.2%	88.8%	
**Sex**				
Male	637 (88.2%)	68 (84.0%)	569 (88.8%)	0.205
Female	85 (11.8%)	13 (16.0%)	72 (11.2%)	
**Age (years)**				
< 65	372 (51.5%)	30 (37.0%)	342 (53.4%)	**0.006**
≥ 65	350 (48.5%)	51 (63.0%)	299 (46.6%)	
**Neoadjuvant treatment**				
No	277 (38.4%)	37 (45.7%)	240 (37.4%)	0.151
Yes	445 (61.6%)	44 (54.3%)	401 (62.6%)	
**(y)pT**				
1	133 (18.4%)	7 (8.6%)	126 (19.7%)	0.105
2	133 (18.4%)	17 (21.0%)	116 (18.1%)	
3	431 (59.7%)	53 (65.4%)	378 (59.0%)	
4	25 (3.5%)	4 (4.9%)	21 (3.3%)	
**(y)pN**				
0	285 (39.5%)	24 (29.6%)	261 (40.8%)	0.221
1	230 (31.9%)	30 (37.0%)	200 (31.3%)	
2	98 (13.6%)	11 (13.6%)	87 (13.6%)	
3	108 (15.0%)	16 (19.8%)	92 (14.4%)	
**L**				
0	307 (42.5%)	31 (38.3%)	276 (43.1%)	0.288
1	273 (37.8%)	37 (45.7%)	236 (36.8%)	
2	142 (19.7%)	13 (16.0%)	129 (20.1%)	
**V**				
0	510 (70.6%)	58 (71.6%)	452 (70.5%)	0.390
1	74 (10.2%)	11 (13.6%)	63 (9.8%)	
2	138 (19.1%)	12 (14.8%)	126 (19.7%)	

The cohort was predominantly male (*n* = 637; 88.2%), with 372 patients (51.5%) aged under 65 years. Pathological staging revealed that 431 patients (59.7%) had (y)pT3 tumors, and 415 (57.5%) presented with lymph node metastasis in the surgical specimen. Regarding treatment, 277 patients (38.4%) underwent primary surgical resection, while 445 (61.6%) received neoadjuvant or perioperative (radio-)chemotherapy.

Loss of SMARCA2 and SMARCA4 was identified in 81 patients (11.2%), of whom 28 cases (34.6%) exhibited heterogeneous SMARCA loss. Cross-table analysis of the entire cohort revealed no significant correlations between SMARCA loss and most clinical parameters, except for age: patients with SMARCA loss were significantly more likely to be older than 64 years of age (63% of the deficient group, Table 1).

### Molecular landscape of SMARCA Loss

The various molecular data collected were available for most, but not all, of the patient cohort. A significant correlation was observed between SMARCA-deficient tumors and a higher frequency of *MET* amplification (16.9% vs. 7.0%, *p* = 0.003, Table 2). This association was particularly pronounced within the neoadjuvant-treated subgroup, where 23.8% of SMARCA-deficient patients harbored a *MET* amplification (*p = 0.002*, Table 3). Other correlations were found: while *TERT* amplification was less frequent across the entire SMARCA-loss population (*p = 0.044*), *PIK3CA* amplification was significantly more prevalent specifically within the primary resected subgroup (*p = 0.015*).

**Table 2:** Correlation between SMARCA expression status (intact vs. deficient) and various molecular and microenvironmental biomarkers in the esophageal adenocarcinoma (EAC) cohort. Biomarkers are categorized by methodology as follows: IHC biomarkers (positive vs. negative expression groups), FISH biomarkers (amplified vs. non-amplified gene copies groups), and CAF density (low vs. high expression groups). *Notes: HER2 positivity was defined as IHC 3 + or IHC 2 + confirmed by FISH amplification; Y-chromosome status was categorized as loss vs. intact expression. Bold print indicates statistical significance.

Biomarkers	SMARCA deficienct (*n* = 81)	SMARCA stable (*n* = 641)	*p*-value
**CAF**			
** Periostin**			
Low	40 (51.9%)	307 (49.8%)	0.727
High	37 (48.1%)	309 (50.2%)	
** FAP**			
Low	39 (48.1%)	313 (50.3%)	0.713
High	42 (51.9%)	309 (49.7%)	
** PDGFRβ**			
Low	34 (43.0%)	320 (51.0%)	0.185
High	45 (57.0%)	308 (49.0%)	
** SMA**			
Low	46 (57.5%)	307 (49.1%)	0.158
High	34 (42.5%)	318 (50.9%)	
**FISH**			
*** TERT***			
None	65 (95.6%)	435 (87.2%)	**0.044**
Amplified	3 (4.4%)	64 (12.8%)	
*** MDM2***			
None	60 (93.8%)	493 (92.8%)	0.789
Amplified	4 (6.3%)	38 (7.2%)	
*** MET***			
None	64 (83.1%)	557 (93.0%)	**0.003**
Amplified	13 (16.9%)	42 (7.0%)	
*** MYC***			
None	62 (78.5%)	497 (80.8%)	0.622
Amplified	17 (21.5%)	118 (19.2%)	
Y-Chromosome*			
Loss (LOY)	30 (47.6%)	297 (58.9%)	0.087
Intact	33 (52.4%)	207 (41.1%)	
*** PIK3CA***			
None	69 (92.0%)	516 (95.4%)	0.210
Amplified	6 (8.0%)	25 (4.6%)	
**IHC**			
** HER2**			
Negative	69 (92.0%)	538 (88.9%)	0.417
Positive*	6 (8.0%)	67 (11.1%)	
** CK5/6**			
Negative	63 (95.5%)	414 (94.7%)	0.806
Positive	3 (4.5%)	23 (5.3%)	
** MTAP**			
Loss	8 (11.0%)	41 (7.3%)	0.268
Intact	65 (89.0%)	522 (92.7%)	
** Claudin 18.2**			
Negative	58 (76.3%)	429 (74.6%)	0.747
Positive	18 (23.7%)	146 (25.4%)	

**Table 3:** Correlation between SMARCA expression status (intact vs. loss) and various tumor microenvironmental and molecular biomarkers, stratified by clinical setting (primary resected patients vs. post-neoadjuvant treatment). Biomarkers are grouped by methodology: CAF quantification (low vs. high expression), FISH biomarkers (amplified vs. non-amplified gene copies; bold values indicate statistical significance), and IHC biomarkers (positive vs. negative expression). Bold print indicates statistical significance.

Biomarkers	Primary resected SMARCA deficient (*n* = 37)	Primary resected SMARCA stable (*n* = 240)	*p*-value	Neoadj. treatment SMARCA deficient (*n* = 44)	Neoadj. treatment SMARCA stable (*n* = 401)	*p*-value
**CAF**						
** Periostin**						
Low	20 (57.1%)	130 (56.3%)	0.923	20 (47.6%)	177 (46.0%)	0.839
High	15 (42.9%)	101 (43.7%)		22 (52.4%)	208 (54.0%)	
** FAP**						
Low	17 (45.9%)	117 (50.4%)	0.612	22 (50.0%)	196 (50.3%)	0.974
High	20 (54.1%)	115 (49.6%)		22 (50.0%)	194 (49.7%)	
** PDGFRβ**						
Low	15 (41.7%)	103 (44.2%)	0.775	19 (44.2%)	217 (54.9%)	0.179
High	21 (58.3%)	130 (55.8%)		24 (55.8%)	178 (45.1%)	
** SMA**						
Low	24 (64.9%)	135 (58.4%)	0.460	22 (51.2%)	172 (43.7%)	0.179
High	13 (35.1%)	96 (41.6%)		21 (48.8%)	222 (56.3%)	
**FISH**						
*** TERT***						
None	33 (97.1%)	170 (90.4%)	0.203	32 (94.1%)	265 (85.2%)	0.154
Amplified	1 (2.9%)	18 (9.6%)		2 (5.9%)	46 (14.8%)	
*** MDM2***						
None	29 (100.0%)	193 (94.6%)	0.200	31 (88.6%)	300 (91.7%)	0.524
Amplified	0 (0.0%)	11 (5.4%)		4 (11.4%)	27 (8.3%)	
*** MET***						
None	32 (91.4%)	216 (96.0%)	0.230	32 (76.2%)	341 (91.2%)	**0.002**
Amplified	3 (8.6%)	9 (4.0%)		10 (23.8%)	33 (8.8%)	
*** MYC***						
None	29 (80.6%)	191 (81.3%)	0.918	33 (76.7%)	306 (80.5%)	0.556
Amplified	7 (19.4%)	44 (18.7%)		10 (23.3%)	74 (19.5%)	
Y-Chromosome*						
Loss (LOY)	12 (40.0%)	107 (56.9%)	0.084	18 (54.5%)	190 (60.1%)	0.534
Intact	18 (60.0%)	81 (43.1%)		15 (45.5%)	126 (39.9%)	
*** PIK3CA***						
None	29 (87.9%)	199 (97.1%)	**0.015**	40 (95.2%)	317 (94.3%)	0.812
Amplified	4 (12.1%)	6 (2.9%)		2 (4.8%)	19 (5.7%)	
*** EGFR***						
None	27 (84.4%)	170 (82.9%)	0.839	37 (88.1%)	263 (79.9%)	0.206
Amplified	5 (15.6%)	35 (17.1%)		5 (11.9%)	66 (20.1%)	
**IHC**						
** HER2**						
Negative	30 (85.7%)	197 (86.0%)	0.961	39 (97.5%)	341 (90.7%)	0.145
Positive*	5 (14.3%)	32 (14.0%)		1 (2.5%)	35 (9.3%)	
** CK5/6**						
Negative	31 (96.9%)	165 (95.4%)	0.704	32 (94.1%)	249 (94.3%)	0.962
Positive	1 (3.1%)	8 (4.6%)		2 (5.9%)	15 (5.7%)	
** MTAP**						
Loss	5 (14.7%)	13 (6.1%)	0.075	3 (7.7%)	28 (8.0%)	0.950
Intact	29 (85.3%)	199 (93.9%)		36 (92.3%)	323 (92.0%)	
** Claudin 18.2**						
Negative	27 (75.0%)	166 (74.1%)	0.909	31 (77.5%)	263 (74.9%)	0.721
Positive	9 (25.0%)	58 (25.9%)		9 (22.5%)	88 (25.1%)	

*Notes: HER2 positivity was defined as IHC 3 + or IHC 2 + confirmed by FISH amplification; Y-chromosome status was categorized as loss vs. intact expression.

### Prognostic markers in SMARCA-intact tumors

**Fig. 1:**
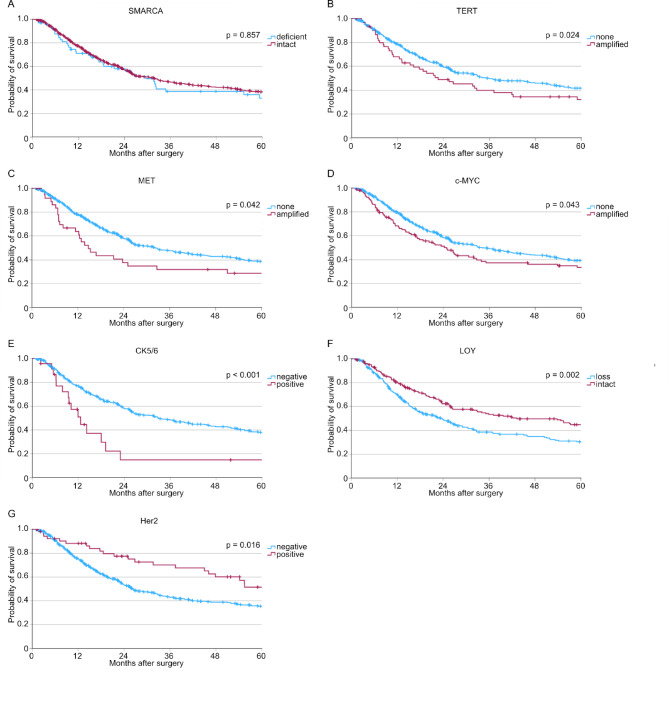
Kaplan-Meier curves for overall survival of the total cohort depending on **(A)** the SMARCA deficiency-status (*p = 0.857*), **(B)** *TERT* amplification-status (*p = 0.024*), **(C)** *MET* amplification-status (*p = 0.042*), **(D)** *MYC* amplification-status (*p = 0.043*), **(E)** CK5/6-expression (= basal subtype of EAC) (*p < 0.001*), **(F)** loss of Y chromosome-status (*p = 0.002*), and **(G)** HER2 expression (*p = 0.016*).

First, we compared the impact of SMARCA-deficiency on patients’ outcomes. Here, no difference between patients with SMARCA-intact and SMARCA-deficient tumors could be observed (*p = 0.857*, Fig. 1A). However, prognostic indicators differed substantially based on SMARCA status. Within the SMARCA-intact group, *TERT* (*p = 0.024*), *MET* (*p = 0.042*) and *MYC* (*p = 0.043*) amplifications, the basal subtype of EAC (= tubular EAC with positivity of basal cytokeratins in ≥ 50% of tumor cells, *p* < 0.001), and LOY (*p = 0.002*) were significantly correlated with diminished overall survival (OS) (Fig. 1B-F). Conversely, HER2 overexpression or amplification was associated with a more favorable prognosis (*p = 0.016*) (Fig. 1G), as published before^19^ In the primary resection subgroup only, TME with accumulation of SMA-positive (*p = 0.031*) CAFs, LOY (*p = 0.044*), as well as the *MYC* amplification (*p* = 0.002), remained associated with poor OS. Additionally, in the neoadjuvant-treated cohort, the loss of the Y chromosome *(p = 0.026)*, the basal subtype (*p < 0.001*), and high FAP expression (*p = 0.025*) in the CAFs were linked to poor survival, while HER2 status (*p = 0.016*) remained a favorable prognostic factor.

### Prognostic markers in SMARCA-deficient tumors

High infiltration of plasma cells (MUM1+) (*p = 0.006*) and mast cells (mast cell tryptase+) (*p = 0.002*), along with high PDGFRβ + CAF expression (*p = 0.048*), were consistently associated with favorable survival. In contrast, LOY (*p = 0.038*) remained an adverse prognostic factor.

**Fig. 2:**
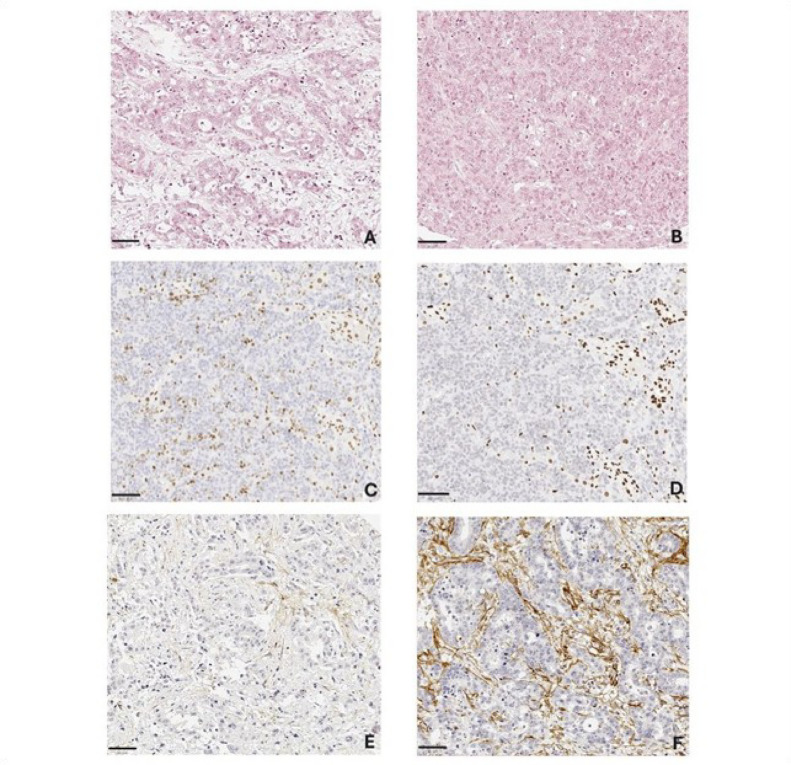
Immunohistochemical staining pattern of fibroblast marker PDGFRb, scale bars 50 μm **(A)** EAC (HE) with tubular growth pattern, **(B)** EAC (HE) with solid growth pattern, **(C)** EAC (IHC) with SMARCA2 loss of expression, **(D)** EAC (IHC) with SMARCA4 loss of expression, EAC (IHC) Platelet derived growth factor receptor beta (PDGFRb) **(E)** low and **(F)** high expression.

### Subanalyses of the SMARCA-deficient cohort

We formed two analysis groups. The first group compares SMARCA-intact with SMARCA-deficient EAC. In this group, the molecular characteristics of the tumors and the composition of CAF subtypes were compared with each other. The second group compares SMARCA-deficient EACs in detail with one another, partly based on growth patterns (tubular versus non-tubular). In this purely SMARCA-deficient group, we also collected detailed data on the inflammatory microenvironment (Tables 4 and 5). Here, SMARCA-deficient tumors showed low ERG-positivity in tumors with a tubular growth pattern (*p = 0.011*, Table 4). All other markers showed a similar distribution in tumors with tubular and non-tubular growth patterns. In tubular SMARCA-deficient tumors, FOXP3+ (*p = 0.029*), MUM1+ (*p = 0.007*), Mast cell tryptase+ cells (*p = 0.033*), and PDGFRβ + cancer-associated fibroblasts (CAFs, Fig. 2) (*p = 0.027*) were linked to improved survival, while *PIK3CA* amplification (*p = 0.014*) predicted poor outcomes. In non-tubular tumors, only MCT expression (*p = 0.039*) was identified as a favorable prognostic marker, while LOY (*p = 0.020*) remained unfavorable.

**Table 4:** Correlation between tubular and non-tubular histology with low/high CAF and TME expression groups on SMARCA-deficient patients. Bold print indicates statistical significance.

Biomarkers (CAF and TME)	Non-tubular Histology (*n* = 40)	Tubular Histology (*n* = 41)	*p* value
**PDGFRb**			
Low	20 (50.0%)	20 (48.8%)	0.913
High	20 (50.0%)	21 (51.2%)	
**Periostin**			
Low	18 (45.0%)	22 (53.7%)	0.436
High	22 (55.0%)	19 (46.3%)	
**Tenascin**			
Low	21 (52.5%)	19 (46.3%)	0.579
High	19 (47.5%)	22 (53.7%)	
**Fap**			
Low	23 (59.0%)	17 (41.5%)	0.117
High	16 (41.0%)	24 (58.5%)	
**SMA**			
Low	24 (61.5%)	23 (56.1%)	0.621
High	15 (38.5%)	18 (43.9%)	
**CD4**			
Low	18 (45.0%)	23 (56.1%)	0.318
High	22 (55.0%)	18 (43.9%)	
**CD8**			
Low	19 (47.5%)	21 (51.2%)	0.738
High	21 (52.5%)	20 (48.8%)	
**CD20**			
Low	23 (57.5%)	18 (43.9%)	0.221
High	17 (42.5%)	23 (56.1%)	
**CD56**			
Low	17 (42.5%)	24 (60.0%)	0.117
High	23 (57.5%)	16 (40.0%)	
**CD66b**			
Low	23 (57.5%)	17 (41.5%)	0.149
High	17 (42.5%)	24 (58.5%)	
**CD68**			
Low	18 (45.0%)	23 (57.5%)	0.263
High	22 (55.0%)	17 (42.5%)	
**CD163**			
Low	16 (40.0%)	25 (61.0%)	0.059
High	24 (60.0%)	16 (39.0%)	
**ERG**			
Low	14 (35.0%)	26 (63.4%)	**0.011**
High	26 (65.0%)	15 (36.6%)	
**FOXP3**			
Low	22 (55.0%)	18 (43.9%)	0.318
High	18 (45.0%)	23 (56.1%)	
**MCT**			
Low	21 (52.5%)	20 (48.8%)	0.738
High	19 (47.5%)	21 (51.2%)	
**MUM1**			
Low	22 (55.0%)	19 (47.5%)	0.502
High	18 (45.0%)	21 (52.5%)	

**Table 5:** Correlation between histological subtype (tubular vs. non-tubular) and various molecular and immunohistochemical biomarkers within the SMARCA-deficient esophageal adenocarcinoma cohort. Biomarkers are categorized by methodology: (b) FISH biomarkers (amplified vs. non-amplified gene copies) and (c) IHC biomarkers (positive vs. negative expression). *Notes: HER2 positivity was defined as IHC 3 + or IHC 2 + confirmed by FISH amplification; Y-chromosome status was categorized as loss vs. intact expression.

Biomarkers	Non-tubular Histology (*n* = 40)	Tubular Histology (*n* = 41)	*p*-value
**FISH**			
*** TERT***			
None	29 (96.7%)	35 (94.6%)	0.683
Amplified	1 (3.3%)	2 (5.4%)	
*** MDM2***			
None	26 (92.9%)	33 (94.3%)	0.817
Amplified	2 (7.1%)	2 (5.7%)	
*** MET***			
None	33 (84.6%)	31 (81.6%)	0.722
Amplified	6 (15.4%)	7 (18.4%)	
*** MYC***			
None	32 (80.0%)	30 (76.9%)	0.739
Amplified	8 (20.0%)	9 (23.1%)	
Y-Chromosome*			
Loss (LOY)	17 (54.8%)	14 (42.4%)	0.321
Intact	14 (45.2%)	19 (57.6%)	
*** PIK3CA***			
None	37 (97.4%)	32 (86.5%)	0.082
Amplified	1 (2.6%)	5 (13.5%)	
*** EGFR***			
None	32 (80.0%)	33 (80.5%)	0.586
Amplified	8 (20.0%)	8 (19.5%)	
**IHC**			
** HER2**			
Negative	35 (87.5%)	34 (82.9%)	0.716
Positive*	5 (12.5%)	7 (17.1%)	
** CK5/6**			
Negative	27 (90.0%)	35 (100.0%)	0.055
Positive	3 (10.0%)	0 (0.0%)	
** MTAP**			
Loss	3 (8.3%)	5 (13.5%)	0.479
Intact	33 (91.7%)	32 (86.5%)	
** Claudin 18.2**			
Negative	26 (68.4%)	32 (82.1%)	0.165
Positive	12 (31.6%)	7 (17.9%)	

### Histopathological characterization between SMARCA-deficient and SMARCA-intact EAC

To further characterize the histopathological spectrum of SMARCA-deficient EAC, all available H&E slides from SMARCA2- and/or SMARCA4-deficient tumors were re-reviewed with particular attention to growth architecture, cytological features, mucin production, necrosis, stromal reaction, and the presence of solid, discohesive, poorly differentiated, or rhabdoid components. Consistent with the predefined classification scheme, tumors were considered non-tubular when any solid, discohesive, or rhabdoid component was present. Many SMARCA-deficient EACs retained conventional adenocarcinoma morphology with irregular infiltrative glandular, tubular, and focally papillary growth. In several cases, luminal necrotic debris compatible with “dirty necrosis” was present within neoplastic glands.

The non-tubular subset showed variable poorly differentiated components, including poorly formed glands, solid nests or sheets, and discohesive single-cell or small-cluster infiltration within a desmoplastic stromal background. These areas were often associated with increased cytological atypia and variable inflammatory infiltrates. However, these features were not uniform across the cohort and did not form a reproducible dominant pattern specific for SMARCA deficiency. One tumor showed prominent extracellular mucin pools with floating neoplastic glandular cell clusters, consistent with mucinous differentiation. Definite recurrent rhabdoid morphology was not identified as a consistent feature.

**Fig. 3:**
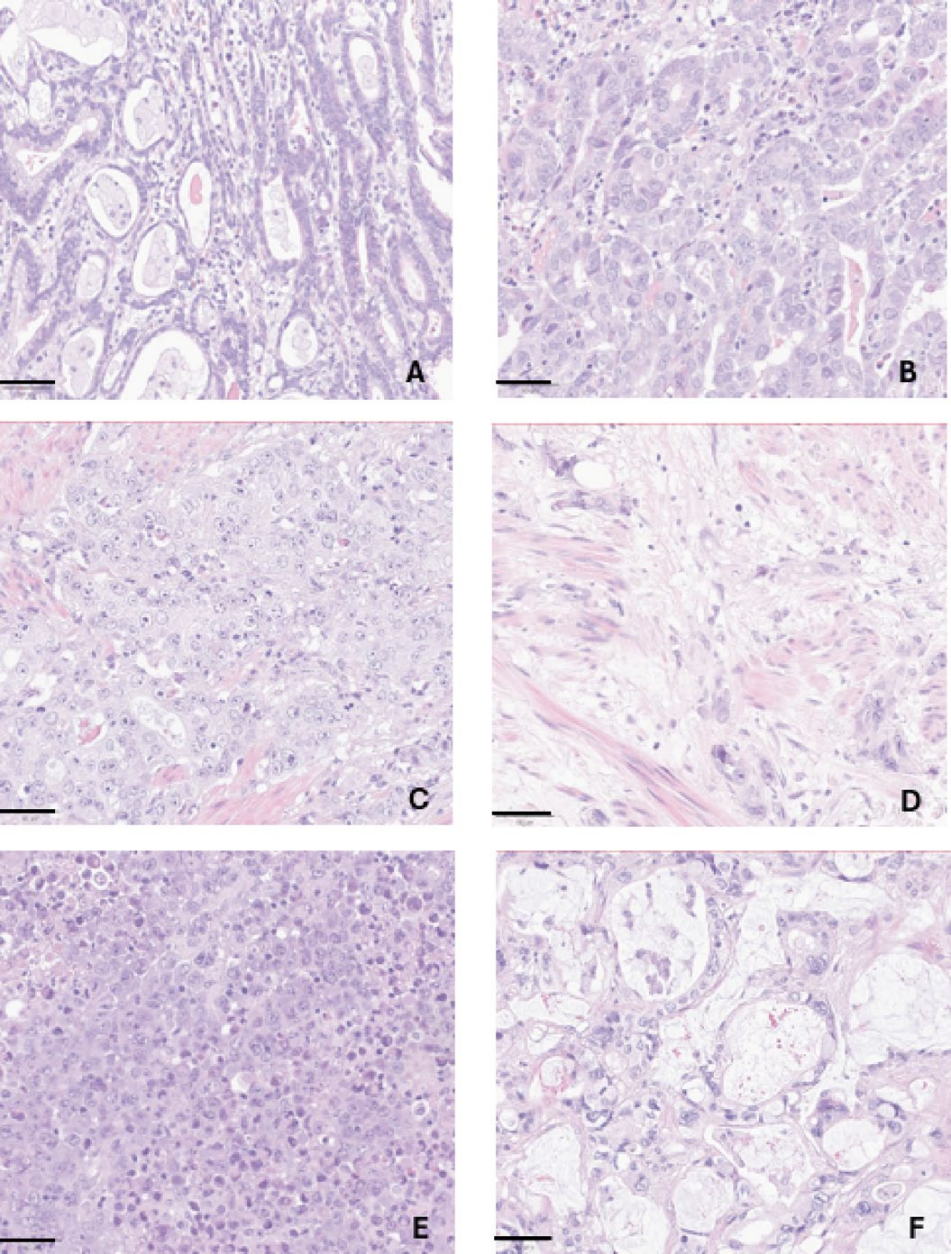
Representative H&E morphology of SMARCA-deficient esophageal adenocarcinoma, scale bars 50 μm **(A)** Conventional gland-forming adenocarcinoma with tubular/papillary architecture; **(B)** tumor with poorly formed glandular structures and focal discohesive growth; **(C)** Focal solid/nested growth pattern with accompanying inflammatory infiltrates; **(D)** discohesive tumor cell infiltration within a desmoplastic stromal background; **(E)** Poorly differentiated solid tumor component with prominent inflammatory background; **(F)** tumor with mucinous differentiation showing prominent extracellular mucin pools with floating neoplastic glandular clusters. Overall, SMARCA-deficient EACs showed a broad histomorphological spectrum encompassing both tubular and non-tubular patterns. Besides conventional gland-forming morphology, a subset of tumors displayed poorly formed glands, solid/nested growth, discohesive infiltration, prominent desmoplastic stroma, and variable inflammatory infiltrates. In some cases, intraluminal necrotic debris compatible with “dirty necrosis” was present. However, no recurrent morphology-specific pattern allowing reliable distinction from SMARCA-intact esophageal adenocarcinoma was identified.

Taken together, SMARCA-deficient EACs in this cohort showed a broad morphological spectrum, ranging from conventional gland-forming tumors to cases with focal or more extensive non-tubular components. Although solid or discohesive growth, dirty necrosis, prominent desmoplasia, inflammatory infiltrates, and occasional mucinous differentiation were observed, we did not identify a recurrent or diagnostically distinctive H&E pattern that reliably separates SMARCA-deficient EAC from SMARCA-intact conventional EAC. These findings support the interpretation that SMARCA deficiency defines a molecular and microenvironmental subset of EAC rather than a strictly morphology-defined histological one (Fig. 3).

### Multivariate analysis

Multivariable Cox Regression analyses showed high CK5-CK6 expression as an independent risk factor for worse patient survival in the total cohort (HR = 2.552, 95% CI = 1.197–5.440, *p* = 0.015; Supplementary Table S6), whereas MTAP intact expression acted as an independent protective factor (HR = 0.509, *p* = 0.020). In the SMARCA-deficient sub-cohort, high *PIK3CA* amplification emerged as a prominent independent risk factor for worse survival (HR = 9.204, 95% CI = 1.680-50.428, *p* = 0.011; Supplementary Table S7). Notably, in this same deficient subset, a preserved, intact Y-chromosome showed a strong clinical trend toward better patient survival (HR = 0.418, *p* = 0.057), marginally crossing the threshold for formal statistical significance.

## Discussion

In this study, we provide a comprehensive clinicopathological, molecular, and tumor microenvironment (TME)-oriented characterization of SMARCA-deficient esophageal adenocarcinoma (EAC) in a large, well-defined Western cohort. Several aspects distinguish the present work from previous reports and underscore its relevance for both tumor biology and clinical stratification of EAC.

First, a major strength of this study is the exceptionally large cohort of 722 Caucasian EAC patients deeply annotated with clinicopathological, molecular, and stromal parameters. To our knowledge, this represents the largest analysis to date addressing the prevalence, composition, and biological implications of SMARCA2/4 loss in EAC. Previous studies on SMARCA-deficient esophageal tumors have largely been limited to small case series, case reports, or mixed cohorts enriched for undifferentiated or rhabdoid carcinomas^37,38^. By contrast, our cohort reflects the full histomorphological spectrum of conventional EAC encountered in Western clinical practice and therefore allows robust conclusions regarding the clinical relevance of this subtype. Within this cohort, SMARCA deficiency was identified in approximately 11% of EACs, confirming that loss of SWI/SNF ATPase activity is not a rare event in this disease^9,38^. Importantly, when restricting the analysis to unequivocally SMARCA-deficient tumors (excluding heterogeneous expression), this subgroup still accounted for nearly 8% of cases, highlighting its biological and potential clinical importance.

Consistent with previous reports on SMARCA2/4-deficient esophageal malignancies, this protein loss was more prevalent among older individuals in our cohort^39,40^.

Second, our data significantly expands current knowledge regarding the molecular landscape of SMARCA-deficient EAC. Among all analyzed oncogenic alterations, *MET* amplification emerged as the most robustly enriched molecular co-alteration in SMARCA-deficient tumors. This association was particularly pronounced in the neoadjuvant-treated subgroup, where nearly one quarter of SMARCA-deficient EACs harbored *MET* amplification^41,42^.

The significant enrichment of *MET* amplification within the SMARCA-deficient subset suggests a non-random molecular association. While the exact mechanistic link in EAC remains to be fully elucidated, similar patterns of extreme aggressiveness and specific molecular co-occurrences have been described in SMARCA4-deficient lung carcinomas^43^. In these models, the loss of SWI/SNF subunits is associated with a profound remodeling of the tumor microenvironment and a dependency on alternative oncogenic pathways. Our data indicate that *MET* amplification might represent one such compensatory mechanism in EAC. Interestingly, this association was not observed for other drivers like HER2 or *EGFR*, suggesting that the synergy between SMARCA deficiency and *MET* signaling may be a specific hallmark of this aggressive subtype, rather than a sign of generalized genomic instability.

*MET* activation may therefore represent a compensatory oncogenic pathway that supports tumor growth and therapy resistance in the context of impaired chromatin remodeling. From a clinical perspective, this observation is highly relevant, as *MET*-amplified EACs have been associated with aggressive behavior and poor outcome, and *MET* represents a potentially druggable target^44^. In addition to *MET*, loss of the Y chromosome (LOY) emerged as a critical adverse factor within SMARCA-deficient tumors. While LOY has previously been described as a frequent event in Barrett-associated adenocarcinoma and linked to poor prognosis, our data demonstrate that its negative prognostic impact persists within the biologically high-risk context of SMARCA deficiency^15,45^. The relationship between our findings and prior investigations on this cohort deserves clarification. While the previous study established LOY as a frequent event in EAC^15^ and the proteomic vulnerabilities of *MET*-amplified cases^16^, our data demonstrate for the first time that these features, *MET* amplification and LOY, are non-randomly enriched within the SMARCA-deficient subset. By shifting the focus from individual markers to a comprehensive ‘SMARCA-deficient’ phenotype, we provide a more integrated molecular model that explains the extreme aggressiveness previously observed in these patients. The combination of SMARCA loss, *MET* amplification, and LOY identifies a subset of EACs with particularly unfavorable clinical behavior, suggesting cooperative effects between chromatin remodeling defects, chromosomal instability, and oncogenic signaling.

Third, the present study provides important insights into the prognostic relevance of SMARCA deficiency itself. SMARCA-deficiency showed no impact on patients’ overall survival. This finding suggests that SMARCA deficiency acts less as an isolated prognostic driver and more as a biological framework that modulates the impact of additional molecular and microenvironmental factors, such as *MET* amplification, LOY, and TME composition. This concept aligns well with emerging data from other tumor entities, where SWI/SNF alterations define aggressive molecular subtypes but require cooperative events to fully determine clinical outcome^46,47^.

Fourth, a particularly novel aspect of this work is the systematic characterization of the inflammatory TME and CAF subtypes in SMARCA-deficient EAC. Overall, CAF composition did not differ substantially between SMARCA-deficient and SMARCA-intact tumors, indicating that SMARCA loss does not globally reprogram stromal abundance. However, within the SMARCA-deficient subgroup, distinct stromal and immune cell populations showed strong prognostic associations. High densities of PDGFRβ-positive CAFs, plasma cells (MUM1+), and mast cells were consistently linked to improved survival, suggesting that specific stromal–immune interactions may partially counterbalance the aggressive biology conferred by SMARCA loss. Conversely, LOY remained a dominant adverse factor across stromal and histological subgroups, underscoring its strong and context-independent prognostic impact. Stratification by histological growth pattern further revealed that SMARCA-deficient tumors are biologically heterogeneous, despite sharing a common chromatin remodeling defect. In tubular SMARCA-deficient EACs, FOXP3 + regulatory T cells and PDGFRβ + CAFs were associated with a favorable outcome, whereas *PIK3CA* amplification identified a poor-prognosis subset. In non-tubular tumors, mast cell infiltration emerged as the only favorable prognostic marker, again highlighting the context-dependent role of the immune microenvironment. These findings suggest that SMARCA deficiency sensitizes tumor behavior to microenvironmental cues, rather than uniformly dictating immune exclusion or stromal activation.

Taken together, our results position SMARCA-deficient EAC as a distinct molecular and biological subtype characterized by (I) frequent co-occurrence with *MET* amplification, (II) LOY with particularly adverse prognostic implications, and (III) a complex, prognostically relevant interplay with specific immune and stromal components. Importantly, the identification of *MET* amplification within this subgroup provides a rational basis for exploring targeted therapeutic strategies, particularly in patients who fail standard multimodal treatment^48^.

All the markers have been selected as they represent the most clinically relevant oncogenic drivers in EAC with potential for targeted therapy. Furthermore, we integrated the analysis of the microenvironmental landscape (CAFs) and large-scale genomic alterations, such as Y-chromosome loss (LOY), to investigate whether SMARCA2/4 deficiency identifies a biologically distinct ‘cluster’ within the heterogeneous landscape of EAC. While mismatch repair (MMR) status and PD-L1 expression are established predictive biomarkers for immunotherapy in gastroesophageal cancers, they were not the primary focus of the present study. Our investigation specifically aimed to characterize the epigenetic dysregulation linked to the SWI/SNF complex and its interaction with tyrosine kinase receptor signaling and the stromal microenvironment. Future prospective studies integrating SMARCA status with MMR/PD-L1 profiles are warranted to better define the immunotherapeutic landscape of this subtype.

Some limitations of this study should be acknowledged. First, this is a retrospective, single-center analysis, which may introduce selection bias despite the large, well-characterized cohort and uniform treatment strategies. Second, SMARCA2 and SMARCA4 status were assessed by immunohistochemistry, which reliably reflects functional loss but does not distinguish between underlying genetic, epigenetic, or post-transcriptional mechanisms; parallel sequencing data were not available for all cases. Third, although tissue microarrays enabled high-throughput and standardized analyses, they may incompletely capture intratumoral heterogeneity of both tumor cells and the tumor microenvironment, despite the use of multi-spot sampling for SMARCA-deficient cases. Fourth, the molecular analyses were limited to selected, clinically relevant gene amplifications and did not include genome-wide or transcriptomic profiling, precluding deeper mechanistic insights.

Another limitation of our study is the assessment of morphology primarily through a tubular/non-tubular classification on TMA cores. This approach may underestimate the presence of focal dedifferentiated or rhabdoid features, which have been described in SMARCA-deficient tumors of other organs. While our large-scale screening using tissue microarrays identifies a molecular subset, further studies on whole-slide images are warranted to provide a more granular histological description of the intratumoral heterogeneity associated with SMARCA2/4 loss.

Due to the exploratory nature of this study on a rare molecular subtype, corrections for multiple statistical testing were not applied to avoid a disproportionate increase in false-negative results. Consequently, marginal p-values should be interpreted with caution and warrant validation in larger, independent cohorts.

In conclusion, this study represents the largest and most comprehensive analysis of SMARCA-deficient EAC to date. By integrating clinicopathological parameters, oncogenic co-alterations, CAF subtypes, and immune infiltration, we demonstrate that SMARCA deficiency is associated with a biologically aggressive EAC subtype whose clinical course is strongly shaped by additional molecular events and the tumor microenvironment. After further characterization and external validation of this subtype, these findings could refine current molecular stratification concepts in EAC and may inform future biomarker-driven therapeutic approaches.

## Supplementary Information

Below is the link to the electronic supplementary material.

Supplementary Material 1

Supplementary Material 2

## Data Availability

The datasets generated and analyzed during the current study are available from the corresponding author on reasonable request.

## Abbreviations

(y)pN: pathological lymph node status (after neoadjuvant therapy)
(y)pT: pathological tumor status (after neoadjuvant therapy)
BRG-1: Brahma-related gene-1
BRM: Brahma homolog
CAF: Cancer-associated fibroblasts
CD: Cluster of differentiation
CEN: Centromere
CEP: Centromere Enumeration Probe
CK: Cytokeratin
*MYC*: cellular Myelocytomatosis
CROSS: Chemoradiotherapy for Oesophageal Cancer Followed by Surgery Study
EAC: esophageal adenocarcinoma
*EGFR*: Epidermal Growth Factor Receptor
ERBB2: Commonly known as HER2
ERG: ETS-related gene
FAP: Fibroblast Activation Protein
FFPE: Formalin-fixed, paraffin-embedded
FISH: fluorescence in situ hybridization
FLOT: Fluorouracil, Leucovorin, Oxaliplatin, Docetaxel
FOXP3: Forkhead box P3
GERD: gastroesophageal reflux disease
HE: Hematoxylin-eosin staining
HER2: Human epidermal growth factor receptor 2
L: lymph vessel infiltration
L0: *no lymphatic vessel infiltration*
L1: *lymphatic vessel infiltration present*
L2: *extensive or diffuse lymphatic vessel*
LOY: Loss of Y-Chromosome
*MDM2*: Mouse double minute
MCT: Mast cell tryptase
*MET*: Mesenchymal-epithelial transition
MMR: Mismatch Repair
MTAP: Methylthioadenosine phosphorylase
MUM1: Interferon regulatory factor 4
PDGFRβ: Platelet-derived growth factor beta
PD-L1: Programmed Death-Ligand 1
*PIK3CA*: Phosphatidylinositol-4,5-bisphosphate 3-kinase catalytic subunit alpha
SCC: Squamous cell carcinoma
SMA: Smooth muscle actin
SMARCA: SWI/SNF-related Matrix-Associated, Actin-dependent regulator of Chromatin A
SWI/SNF: Switch/Sucrose Non-Fermentable, ATP-dependent remodelling complexes
*TERT*: Telomerase Reverse Transcriptase
TMA: Tissue microarray
TME: Tumor microenvironment
V: blood vessel infiltration
V0: *no blood vessel infiltration*
V1: *microscopic blood vessel infiltration*
V2: *macroscopic blood vessel infiltration*
