# Supplementary material for: SMARCA2 and SMARCA4-deficiency is associated with a distinct molecular and microenvironmental subtype of esophageal adenocarcinoma
Source: Sci Rep. 2026 Jul 30;16:23610. doi: 10.1038/s41598-026-60346-8 (PMC13421451; doi:10.1038/s41598-026-60346-8)
Supplement: Supplementary file 1 — Supplementary Material 1 [file 41598_2026_60346_MOESM1_ESM.docx]

| **Clinicopathological features** | **SMARCA2/SMARCA4**  **Co-deficienct**  **(n = 10)** | **All the rest**  **(n = 712)** | ***p-value*** |
| --- | --- | --- | --- |
| **Sex** |  |  |  |
| Male | 9 (90.0%) | 628 (88.2%) | 0.861 |
| Female | 1 (10.0%) | 84 (11.8%) |  |
| **Age (years)** |  |  |  |
| < 65 | 4 (40.0%) | 368 (51.7%) | 0.463 |
| ≥ 65 | 6 (60.0%) | 344 (48.3%) |  |
| **Neoadjuvant treatment** |  |  |  |
| No | 5 (50.0%) | 272 (38.2%) | 0.446 |
| Yes | 5 (50.0%) | 440 (61.8%) |  |
| **(y)pT** |  |  |  |
| 1 | 2 (20.0%) | 131 (18.4%) | 0.696 |
| 2 | 2 (20.0%) | 131 (18.4%) |  |
| 3 | 5 (50.0%) | 426 (59.8%) |  |
| 4 | 1 (10.0%) | 24 (3.4%) |  |
| **(y)pN** |  |  |  |
| 0 | 4 (40.0%) | 281 (39.5%) | 0.964 |
| 1 | 3 (30.0%) | 227 (31.9%) |  |
| 2 | 1 (10.0%) | 97 (13.6%) |  |
| 3 | 2 (20.0%) | 106 (14.9%) |  |
| **L** |  |  |  |
| 0 | 3 (30.0%) | 304 (42.7%) | 0.679 |
| 1 | 5 (50.0%) | 268 (37.6%) |  |
| 2 | 2 (20.0%) | 140 (19.7%) |  |
| **V** |  |  |  |
| 0 | 8 (80.0%) | 502 (70.5%) | 0.557 |
| 1 | 0 (0.0%) | 74 (10.4%) |  |
| 2 | 2 (20.0%) | 136 (19.1%) |  |
|  |  |  |  |

Supplementary Table S3 - Clinicopathological characteristics of the esophageal adenocarcinoma cohort: comparison between SMARCA2/SMARCA4 co-deficient cases and the remaining cohort. (y)pN: pathological lymph node status (after neoadjuvant therapy), (y)pT: pathological tumor status (after neoadjuvant therapy), L0: No lymphatic vessel infiltration, L1: lymphatic vessel infiltration present, L2: extensive or diffuse lymphatic vessel, V0: No blood vessel infiltration, V1 microscopic blood vessel infiltration, V2: macroscopic blood vessel infiltration.

| **Biomarkers** | **SMARCA2/SMARCA4**  **Co-deficienct (n = 10)** | **All the rest**  **(n = 712)** | ***p-value*** |
| --- | --- | --- | --- |
| **CAF** |  |  |  |
| **Periostin** |  |  |  |
| Low | 7 (70.0%) | 340 (49.8%) | 0.204 |
| High | 3 (30.0%) | 343 (50.2%) |  |
| **FAP** |  |  |  |
| Low | 6 (60.0%) | 346 (49.9%) | 0.527 |
| High | 4 (40.0%) | 347 (50.1%) |  |
| **PDGFRβ** |  |  |  |
| Low | 2 (22.2%) | 352 (50.4%) | 0.093 |
| High | 7 (77.8%) | 346 (49.6%) |  |
| **SMA** |  |  |  |
| Low | 6 (66.7%) | 347 (49.9%) | 0.316 |
| High | 3 (33.3%) | 349 (50.1%) |  |
| **FISH** |  |  |  |
| ***TERT*** |  |  |  |
| None | 7 (100.0%) | 493 (88.0%) | 0.330 |
| Amplified | 0 (0.0%) | 67 (12.0%) |  |
| ***MDM2*** |  |  |  |
| None | 9 (100.0%) | 544 (92.8%) | 0.405 |
| Amplified | 0 (0.0%) | 42 (7.2%) |  |
| ***MET*** |  |  |  |
| None | 8 (80.0%) | 613 (92.0%) | 0.167 |
| Amplified | 2 (20.0%) | 53 (8.0%) |  |
| ***MYC*** |  |  |  |
| None | 9 (90.0%) | 550 (80.4%) | 0.447 |
| Amplified | 1 (10.0%) | 134 (19.6%) |  |
| **Y-Chromosome*** |  |  |  |
| loss (LOY) | 5 (62.5%) | 322 (57.6%) | 0.781 |
| intact | 3 (37.5%) | 237 (42.4%) |  |
| ***PIK3CA*** |  |  |  |
| None | 10 (100.0%) | 575 (94.9%) | 0.463 |
| Amplified | 0 (0.0%) | 31 (5.1%) |  |
| ***EGFR*** |  |  |  |
| None | 9 (100.0%) | 488 (81.5%) | 0.153 |
| Amplified | 0 (0.0%) | 111 (18.5%) |  |
| **IHC** |  |  |  |
| **HER2** |  |  |  |
| Negative | 9 (100.0%) | 598 (89.1%) | 0.295 |
| Positive* | 0 (0.0%) | 73 (10.9%) |  |
| **CK5/6** |  |  |  |
| Negative | 9 (100.0%) | 468 (94.7%) | 0.480 |
| Positive | 0 (0.0%) | 26 (5.3%) |  |
| **MTAP** |  |  |  |
| loss | 1 (12.5%) | 48 (7.6%) | 0.609 |
| intact | 7 (87.5%) | 580 (92.4%) |  |
| **Claudin 18.2** |  |  |  |
| Negative | 8 (80.0%) | 479 (74.7%) | 0.703 |
| Positive | 2 (20.0%) | 162 (25.3%) |  |

Supplementary Table S3a - Correlation between SMARCA2/SMARCA4 co-deficient cases and the remaining cohort on various molecular and microenvironmental biomarkers in the esophageal adenocarcinoma (EAC) cohort. Biomarkers are categorized by methodology as follows: IHC biomarkers (positive vs. negative expression groups), FISH biomarkers (amplified vs. non-amplified gene copies groups), and CAF density (low vs. high expression groups).*Notes: HER2 positivity was defined as IHC 3+ or IHC 2+ confirmed by FISH amplification; Y-chromosome status was categorized as loss vs. intact expression.

| **Clinicopathological features** | **SMARCA2 deficiency**  **(n = 73)** | **All the rest**  **(n = 649)** | ***p-value*** |
| --- | --- | --- | --- |
| **Sex** |  |  |  |
| Male | 60 (82.2%) | 568 (88.8%) | 0.101 |
| Female | 13 (17.8%) | 72 (11.3%) |  |
| **Age (years)** |  |  |  |
| < 65 | 29 (39.7%) | 338 (52.8%) | **0.034** |
| ≥ 65 | 44 (60.3%) | 302 (47.2%) |  |
| **Neoadjuvant treatment** |  |  |  |
| No | 32 (43.8%) | 243 (38.0%) | 0.329 |
| Yes | 41 (56.2%) | 397 (62.0%) |  |
| **(y)pT** |  |  |  |
| 1 | 5 (6.8%) | 126 (19.7%) | 0.064 |
| 2 | 16 (21.9%) | 114 (17.8%) |  |
| 3 | 49 (67.1%) | 378 (59.1%) |  |
| 4 | 3 (4.1%) | 22 (3.4%) |  |
| **(y)pN** |  |  |  |
| 0 | 21 (28.8%) | 262 (41.0%) | 0.158 |
| 1 | 29 (39.7%) | 195 (30.5%) |  |
| 2 | 9 (12.3%) | 88 (13.8%) |  |
| 3 | 14 (19.2%) | 94 (14.7%) |  |
| **L** |  |  |  |
| 0 | 26 (35.6%) | 277 (43.3%) | 0.284 |
| 1 | 34 (46.6%) | 238 (37.2%) |  |
| 2 | 13 (17.8%) | 125 (19.5%) |  |
| **V** |  |  |  |
| 0 | 51 (69.9%) | 454 (70.9%) | 0.573 |
| 1 | 10 (13.7%) | 64 (10.0%) |  |
| 2 | 12 (16.4%) | 122 (19.1%) |  |

Supplementary Table S4 - Clinicopathological characteristics of the esophageal adenocarcinoma cohort: comparison between SMARCA2 deficient cases and the remaining cohort. (y)pN: pathological lymph node status (after neoadjuvant therapy), (y)pT: pathological tumor status (after neoadjuvant therapy), L0: No lymphatic vessel infiltration, L1: lymphatic vessel infiltration present, L2: extensive or diffuse lymphatic vessel, V0: No blood vessel infiltration, V1 microscopic blood vessel infiltration, V2: macroscopic blood vessel infiltration

| **Biomarkers** | **SMARCA2 deficiency (n = 73)** | **All the rest**  **(n = 649)** | ***p-value*** |
| --- | --- | --- | --- |
| **CAF** |  |  |  |
| **Periostin** |  |  |  |
| Low | 34 (49.3%) | 310 (50.2%) | 0.879 |
| High | 35 (50.7%) | 307 (49.8%) |  |
| **FAP** |  |  |  |
| Low | 37 (50.7%) | 310 (49.8%) | 0.891 |
| High | 36 (49.3%) | 312 (50.2%) |  |
| **PDGFRβ** |  |  |  |
| Low | 30 (42.3%) | 320 (51.0%) | 0.165 |
| High | 41 (57.7%) | 308 (49.0%) |  |
| **SMA** |  |  |  |
| Low | 40 (55.6%) | 309 (49.4%) | 0.326 |
| High | 32 (44.4%) | 316 (50.6%) |  |
| **FISH** |  |  |  |
| ***TERT*** |  |  |  |
| None | 58 (95.1%) | 438 (87.3%) | 0.074 |
| Amplified | 3 (4.9%) | 64 (12.7%) |  |
| ***MDM2*** |  |  |  |
| None | 54 (93.1%) | 493 (92.8%) | 0.942 |
| Amplified | 4 (6.9%) | 38 (7.2%) |  |
| ***MET*** |  |  |  |
| None | 56 (81.2%) | 559 (93.0%) | **<0.001** |
| Amplified | 13 (18.8%) | 42 (7.0%) |  |
| ***MYC*** |  |  |  |
| None | 55 (77.5%) | 497 (80.7%) | 0.518 |
| Amplified | 16 (22.5%) | 119 (19.3%) |  |
| **Y-Chromosome*** |  |  |  |
| loss (LOY) | 27 (48.2%) | 298 (59.0%) | 0.121 |
| intact | 29 (51.8%) | 207 (41.0%) |  |
| ***PIK3CA*** |  |  |  |
| None | 65 (94.2%) | 514 (95.0%) | 0.774 |
| Amplified | 4 (5.8%) | 27 (5.0%) |  |
| ***EGFR*** |  |  |  |
| None | 59 (86.8%) | 434 (81.1%) | 0.256 |
| Amplified | 9 (13.2%) | 101 (18.9%) |  |
| **IHC** |  |  |  |
| **HER2** |  |  |  |
| Negative | 63 (91.3%) | 541 (89.1%) | 0.578 |
| Positive* | 6 (8.7%) | 66 (10.9%) |  |
| **CK5/6** |  |  |  |
| Negative | 58 (96.7%) | 416 (94.8%) | 0.526 |
| Positive | 2 (3.3%) | 23 (5.2%) |  |
| **MTAP** |  |  |  |
| loss | 8 (12.3%) | 41 (7.2%) | 0.149 |
| intact | 57 (87.7%) | 525 (92.8%) |  |
| **Claudin 18.2** |  |  |  |
| Negative | 51 (73.9%) | 432 (74.9%) | 0.863 |
| Positive | 18 (26.1%) | 145 (25.1%) |  |
|  |  |  |  |
|  |  |  |  |

Supplementary Table S4a - Correlation between SMARCA2 deficient cases and the remaining cohort on various molecular and microenvironmental biomarkers in the oesophageal adenocarcinoma (EAC) cohort. Biomarkers are categorized by methodology as follows: IHC biomarkers (positive vs. negative expression groups), FISH biomarkers (amplified vs. non-amplified gene copies groups), and CAF density (low vs. high expression groups).*Notes: HER2 positivity was defined as IHC 3+ or IHC 2+ confirmed by FISH amplification; Y-chromosome status was categorized as loss vs. intact expression.

| **Clinicopathological features** | **SMARCA4 deficiency**  **(n = 18)** | **All the rest**  **(n = 704)** | ***p-value*** |
| --- | --- | --- | --- |
| **Sex** |  |  |  |
| Male | 17 (94.4%) | 620 (88.1%) | 0.407 |
| Female | 1 (5.6%) | 84 (11.9%) |  |
| **Age (years)** |  |  |  |
| < 65 | 5 (27.8%) | 367 (52.1%) | 0.041 |
| ≥ 65 | 13 (72.2%) | 337 (47.9%) |  |
| **Neoadjuvant treatment** |  |  |  |
| No | 10 (55.6%) | 267 (37.9%) | 0.129 |
| Yes | 8 (44.4%) | 437 (62.1%) |  |
| **(y)pT** |  |  |  |
| 1 | 4 (22.2%) | 129 (18.3%) | 0.310 |
| 2 | 3 (16.7%) | 130 (18.5%) |  |
| 3 | 9 (50.0%) | 422 (59.9%) |  |
| 4 | 2 (11.1%) | 23 (3.3%) |  |
| **(y)pN** |  |  |  |
| 0 | 7 (38.9%) | 278 (39.5%) | 0.725 |
| 1 | 4 (22.2%) | 226 (32.1%) |  |
| 2 | 3 (16.7%) | 95 (13.5%) |  |
| 3 | 4 (22.2%) | 104 (14.8%) |  |
| **L** |  |  |  |
| 0 | 8 (44.4%) | 299 (42.5%) | 0.632 |
| 1 | 8 (44.4%) | 265 (37.6%) |  |
| 2 | 2 (11.1%) | 140 (19.9%) |  |
| **V** |  |  |  |
| 0 | 15 (83.3%) | 495 (70.3%) | 0.488 |
| 1 | 1 (5.6%) | 73 (10.4%) |  |
| 2 | 2 (11.1%) | 136 (19.3%) |  |

Supplementary Table S5 - Clinicopathological characteristics of the oesophageal adenocarcinoma cohort: comparison between SMARCA4 deficient cases and the remaining cohort. (y)pN: pathological lymph node status (after neoadjuvant therapy), (y)pT: pathological tumor status (after neoadjuvant therapy), L0: No lymphatic vessel infiltration, L1: lymphatic vessel infiltration present, L2: extensive or diffuse lymphatic vessel, V0: No blood vessel infiltration, V1 microscopic blood vessel infiltration, V2: macroscopic blood vessel infiltration.

| **Biomarkers** | **SMARCA4 deficiency (n = 18)** | **All the rest**  **(n = 704)** | ***p-value*** |
| --- | --- | --- | --- |
| **CAF** |  |  |  |
| **Periostin** |  |  |  |
| Low | 13 (72.2%) | 334 (49.5%) | 0.057 |
| High | 5 (27.8%) | 341 (50.5%) |  |
| **FAP** |  |  |  |
| Low | 8 (44.4%) | 344 (50.2%) | 0.629 |
| High | 10 (55.6%) | 341 (49.8%) |  |
| **PDGFRβ** |  |  |  |
| Low | 6 (35.3%) | 348 (50.4%) | 0.217 |
| High | 11 (64.7%) | 342 (49.6%) |  |
| **SMA** |  |  |  |
| Low | 12 (70.6%) | 341 (49.6%) | 0.087 |
| High | 5 (29.4%) | 347 (50.4%) |  |
| **FISH** |  |  |  |
| ***TERT*** |  |  |  |
| None | 14 (100.0%) | 486 (87.9%) | 0.165 |
| Amplified | 0 (0.0%) | 67 (12.1%) |  |
| ***MDM2*** |  |  |  |
| None | 15 (100.0%) | 538 (92.8%) | 0.280 |
| Amplified | 0 (0.0%) | 42 (7.2%) |  |
| ***MET*** |  |  |  |
| None | 16 (88.9%) | 605 (91.9%) | 0.640 |
| Amplified | 2 (11.1%) | 53 (8.1%) |  |
| ***MYC*** |  |  |  |
| None | 16 (88.9%) | 543 (80.3%) | 0.365 |
| Amplified | 2 (11.1%) | 133 (19.7%) |  |
| **Y-Chromosome*** |  |  |  |
| loss (LOY) | 8 (53.3%) | 319 (57.8%) | 0.730 |
| intact | 7 (46.7%) | 233 (42.2%) |  |
| ***PIK3CA*** |  |  |  |
| None | 14 (87.5%) | 571 (95.2%) | 0.166 |
| Amplified | 2 (12.5%) | 29 (4.8%) |  |
| ***EGFR*** |  |  |  |
| None | 14 (93.3%) | 483 (81.5%) | 0.239 |
| Amplified | 1 (6.7%) | 110 (18.5%) |  |
| **IHC** |  |  |  |
| **HER2** |  |  |  |
| Negative | 15 (100.0%) | 592 (89.0%) | 0.174 |
| Positive* | 0 (0.0%) | 73 (11.0%) |  |
| **CK5/6** |  |  |  |
| Negative | 14 (93.3%) | 463 (94.9%) | 0.790 |
| Positive | 1 (6.7%) | 25 (5.1%) |  |
| **MTAP** |  |  |  |
| loss | 1 (6.3%) | 48 (7.7%) | 0.825 |
| intact | 15 (93.8%) | 572 (92.3%) |  |
| **Claudin 18.2** |  |  |  |
| Negative | 15 (88.2%) | 472 (74.4%) | 0.196 |
| Positive | 2 (11.8%) | 162 (25.6%) |  |

Supplementary Table S5a - Correlation between SMARCA4 deficient cases and the remaining cohort on various molecular and microenvironmental biomarkers in the oesophageal adenocarcinoma (EAC) cohort. Biomarkers are categorized by methodology as follows: IHC biomarkers (positive vs. negative expression groups), FISH biomarkers (amplified vs. non-amplified gene copies groups), and CAF density (low vs. high expression groups).*Notes: HER2 positivity was defined as IHC 3+ or IHC 2+ confirmed by FISH amplification; Y-chromosome status was categorized as loss vs. intact expression.

| **Variables** | **Hazard Ratio (95% CI)** | ***p-value*** |
| --- | --- | --- |
| **Age group** (> 65 yrs vs. < 65 yrs) | 1.479 (1.034–2.117) | **0.032** |
| **Neoadjuvant treatment** (No vs. Yes) | 1.028 (0.680–1.554) | 0.897 |
| **pT** (pT3/4 vs. pT1/2) | 1.585 (1.172–2.143) | **0.003** |
| **pN** (pN+ vs. pN0) | 1.592 (1.359–1.864) | **<0.001** |
| **V** (V+ vs. V0) | 0.811 (0.632–1.041) | 0.100 |
| **FISH (Fluorescence In Situ Hybridization)** |  |  |
| ***TERT*** (amplified vs. none) | 0.794 (0.487–1.296) | 0.357 |
| ***MET*** (amplified vs. none) | 0.886 (0.475–1.654) | 0.705 |
| ***MYC*** (amplified vs. none) | 0.942 (0.612–1.451) | 0.786 |
| **Y-Chromosome** (intact vs. loss) | 0.760 (0.531–1.089) | 0.135 |
| **IHC (Immunohistochemistry)** |  |  |
| **HER2** (positive vs. negative) | 0.794 (0.431–1.465) | 0.461 |
| **CK5/6** (positive vs. negative) | 2.552 (1.197–5.440) | **0.015** |
| **MTAP** (intact vs. loss) | 0.509 (0.289–0.898) | **0.020** |
| **CAF (Cancer-Associated Fibroblasts)** |  |  |
| **FAP** (high expression vs. low expression) | 0.889 (0.625–1.265) | 0.514 |
| **SMA** (high expression vs. low expression) | 1.040 (0.725–1.492) | 0.831 |

Supplementary Table S6 - Multivariable Cox Regression analyses for the total cohort

| **Variables** | **Hazard Ratio (95% CI)** | ***p-value*** |
| --- | --- | --- |
| **pN** (pN+ vs. pN0) | 1.640 (1.056–2.545) | **0.027** |
| **FISH (Fluorescence In Situ Hybridization)** |  |  |
| **Y-Chromosome** (intact vs. loss) | 0.418 (0.171–1.025) | 0.057 |
| ***PIK3CA*** (amplified vs. none) | 9.204 (1.680–50.428) | **0.011** |
| **CAF (Cancer-Associated Fibroblasts)** |  |  |
| **PDGFRβ** (high expression vs. low expression) | 0.677 (0.290–1.578) | 0.366 |

Supplementary Table S7 - Multivariable Cox Regression analyses for SMARCA deficient cohort

*
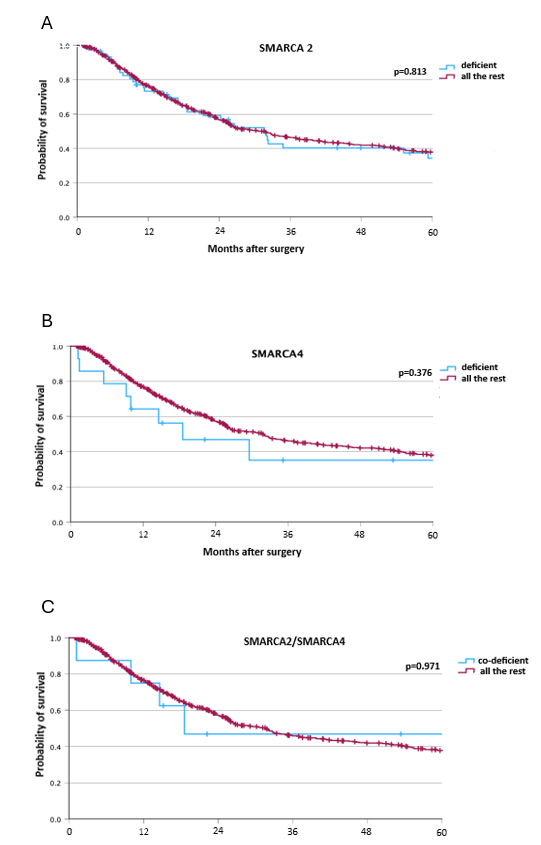
*

Figure S1. Kaplan-Meier survival curves for overall survival (OS) of patients with esophageal adenocarcinoma (EAC) stratified by SMARCA expression status. The prognostic impact on overall survival is shown for: (A) SMARCA2 deficiency status (p = 0.813), (B) SMARCA4 deficiency status (p = 0.376), and (C) SMARCA2/SMARCA4 co-deficiency status (p = 0.971).
