# Supplementary material for: SMARCA2 and SMARCA4-deficiency is associated with a distinct molecular and microenvironmental subtype of esophageal adenocarcinoma
Source: Sci Rep. 2026 Jul 30;16:23610. doi: 10.1038/s41598-026-60346-8 (PMC13421451; doi:10.1038/s41598-026-60346-8)
Supplement: Supplementary file 2 — Supplementary Material 2 [file 41598_2026_60346_MOESM2_ESM.docx]

| **Antibody** | **Manufacturer** | **Company Headquarters** | **Clone** | **Dilution** | **Pretreatment** | **Order Number** |
| --- | --- | --- | --- | --- | --- | --- |
| **CD4** | Quartett | Berlin, Germany | QR032, rabbit | 1:100 | EDTA | C-C012-10 |
| **CD8** | Dako (Agilent) | Glostrup, Denmark | C8/144B, mouse | 1:200 | Citrate | M7103 |
| **CD20** | Dako (Agilent) | Glostrup, Denmark | L26, mouse | 1:1250 | Citrate | M0755 |
| **CD56** | Thermo Fisher Scientific | Waltham, MA, USA | 123C3 | 1:500 | EDTA | MA5-16445 |
| **CD66b** | Novus Biologicals | Centennial, CO, USA | G10F5, mouse | 1:200 | EDTA | NB100-77808 |
| **CD68** | Dako (Agilent) | Glostrup, Denmark | PG-M1, mouse | 1:400 | EDTA | M0876 |
| **CD163** | Cell Marque | Rocklin, CA, USA | MRQ-26, mouse | 1:100 | EDTA | 163M-16 |
| **FOXP3** | Abcam | Cambridge, UK | 236A/E7, mouse | 1:100 | EDTA | ab20034 |
| **Mast Cell Tryptase** | Dako (Agilent) | Glostrup, Denmark | AA1, mouse | 1:4000 | Enzyme | M7052 |
| **MUM1** | Dako (Agilent) | Glostrup, Denmark | MUM1p, mouse | 1:1000 | Citrate | M7259 |
| **ERG** | Cell Marque | Rocklin, CA, USA | EP111, rabbit | 1:300 | EDTA | 434R-15 |
| **FAP** | Abcam | Cambridge, UK | EPR20021 | 1:200 | Citrate | ab207178 |
| **Periostin** | Abcam | Cambridge, UK | EPR19934 | 1:2000 | EDTA | ab219056 |
| **PDGFRβ** | Abcam | Cambridge, UK | Y92, rabbit | 1:300 | EDTA | ab32570 |
| **SMA** | Dako (Agilent) | Glostrup, Denmark | 1A4, mouse | 1:4000 | None | M0851 |
| **Tenascin** | Abcam | Cambridge, UK | EPR4219, rabbit | 1:400 | Citrate | ab1089 |
| **CK5/6** | Cell Marque | Rocklin, CA, USA | D5 & 16B4, mouse | 1:50 | EDTA | 356M |
| **BRG1 (SMARCA4)** | Abcam | Cambridge, UK | EPNCIR111A, rabbit | 1:300 | EDTA | ab110641 |
| **BRM (SMARCA2)** | Cell Signaling Technology | Danvers, MA, USA | D9E8B, rabbit | 1:50 | EDTA | 11966S |
| **Claudin 18.2** | Roche Tissue Diagnostics | Tucson, AZ, USA | 43-14A, mouse | Ready-to-use | EDTA | 8504148001 |
| **HER2** | Roche Tissue Diagnostics | Tucson, AZ, USA | 4B5, rabbit | Ready-to-use | EDTA | 790-2991 |
| **MTAP** | Cell Signaling Technology | Danvers, MA, USA | E5R1I, rabbit | 1:1000 | EDTA | 74683S |

**Supplementary Table S1 – Detailed IHC antibody information**

| **Target Locus / Biomarker** | **Manufacturer** | **Company Headquarters** | **FISH Probe Details** |
| --- | --- | --- | --- |
| ***TERT*** | Zytomed Systems | Berlin, Germany | SPEC TERT / 5q31 Dual Color Probe |
| ***MDM2*** | Zytomed Systems | Berlin, Germany | SPEC MDM2 / CEN 12 Dual Color Probe |
| ***MET*** | Zytomed Systems | Berlin, Germany | SPEC MET / CEN 7 Dual Color Probe |
| ***MYC*** | Zytomed Systems | Berlin, Germany | SPEC MYC / CEN 8 Dual Color Probe |
| ***PIK3CA*** | Zytomed Systems | Berlin, Germany | SPEC PIK3CA / CEN 3 Dual Color Probe |
| ***EGFR*** | Zytomed Systems | Berlin, Germany | SPEC EGFR / CEN 7 Dual Color Probe |
| **Y-Chromosome** | Abbott Molecular | Wiesbaden, Germany | Vysis Yq12 / Yp11.3 Satellite Enumeration |

**Supplementary Table S2 - Detailed FISH antibody information**
